# Identification of Bernalite Transformation and Tridentate Arsenate Complex at Nano-goethite under Effects of Drying, pH and Surface Loading

**DOI:** 10.1038/s41598-018-26808-4

**Published:** 2018-05-30

**Authors:** Junho Han, Hee-Myong Ro

**Affiliations:** Department of Agricultural Biotechnology and Research Institute of Agriculture and Life Sciences, Seoul National University, Seoul, 08826 Republic of Korea

## Abstract

The structural configuration of arsenate on iron (hydr)oxide determines its leachability and bioavailability in the soil environment. It is important to understand how the stability of iron hydroxide and the structural configuration of arsenate complexes vary in response to changes in environmental conditions. Therefore, we investigated the effects of drying, pH and surface loadings on the stability of goethite and the structural configuration of arsenate through batch experiments and TEM and XAS measurements with DFT calculation. As a result, we observed no significant transformation of goethite under most conditions, but TEM confirmed the partial formation of bernalite in the presence of arsenate at a pH of 10, and the bernalite showed 2.18 times higher arsenate sorption than the goethite. The linear combination fitting of the EXAFS spectra with DFT calculations revealed that tridentate and bidentate complexes were dominant under low surface loading and pH conditions in the sedimented samples, while monodentate complexes were abundant under high surface loading and pH conditions. Based on our results, we conclude that the formation of arsenic-rich colloids could account for mobilization in the soil environment, and the density of available sorption sites combined with the concentration of solute could cause the change in structural configuration.

## Introduction

Arsenic is one of the most notorious elements on Earth due to its abundance, toxicity and usage worldwide^1,2^. Over 20 countries have suffered from groundwater contamination by arsenic originating from both natural and anthropogenic sources, such as mining, agricultural chemicals and wood preservatives^3,4^, which have accelerated arsenic contamination in the soil environment^4–6^. Arsenic accumulation poses a serious threat to the health of humans and ecosystems^1,2,7,8^, as arsenate (the major oxyanion form of arsenic) resembles phosphate and can block phosphate metabolism, thus causing various problems in humans^7,9,10^. However, it is extremely difficult to counteract such problems due to the distinctive dynamics of arsenic in the soil environment, such as its species transformation, methylation, transport, precipitation and adsorption^2,11,12^.

Soil is a heterogeneous aggregate of various materials, such as phyllosilicates, metal (hydr)oxides and organic matter. Among them, nanosized iron (hydr)oxides have been reported to be the key component in retaining oxyanions due to their relative abundance (as Fe is the 4^th^ most abundant element), high surface area and charged surface^13,14^. In addition, their crystal structure and morphology also determine their characteristics and govern the arsenate dynamics in the soil environment^15–17^. It is not only the sorbent characteristics but also the interactions between the arsenate and sorbent that are important. There are two main types of interactions: the inner-sphere complex is formed by adsorption via covalent bonds, while the outer-sphere complex is formed by adsorption via electrostatic attraction, dispersion interactions and hydrophobic effects. In addition, the inner-sphere complex can be monodentate, bidendate or tridentate, and the structural configuration of the inner-sphere complex is essential because it determines the leachability and bioavailability of the arsenate in the soil environment^18–21^.

Numerous studies have attempted to reveal the structural configuration of arsenate in iron oxides, but it still remains controversial. A bidentate binuclear (BB) complex has been confirmed to be the major structural con-figuration of arsenate on iron oxides^22–29^, but few studies have also identified bidentate mononuclear (BM)^22,28^ and monodentate mononuclear (MM) complexes^21,22,25^. Previous studies have confirmed the transition of this structural configuration with different environmental conditions; for example, Elzinga and Sparks^30^, Waychunas *et al*.^25^, He *et al*.^31^ and Abdala *et al*.^32^ reported the effects of pH and surface loading on this transition, while Gu *et al*.^33^ reported the effects of drying. From the literature, it is clear that the transition of the structural configuration is highly dependent on environmental conditions, but the inter-connections between environmental conditions have not yet been fully addressed.

In addition, the interpretation of the adsorbed arsenate using the extended X-ray absorption fine structure (EXAFS) technique is quite challenging because it has a relatively low signal-to-noise ratio and it is extremely difficult to construct a model structure. For that reason, most studies have employed scorodite as a model structure for the shell fitting analysis. However, when we carefully reviewed the previous studies, we observed a slight peak shift around 2.4–2.7 Å by changing pH, surface loading and competition from the shell fitting results with the scorodite; these studies reported that multiple scattering was the reason for this shift^21,27,28^. However, we observed the decreasing residual of fit with increasing pH and surface loading, perhaps indicating that an unknown complex may overlap with the multiple scattering. In addition, recent studies also reported the possibility of a tridentate complex. Auffan *et al*.^34^ and Liu *et al*.^35^ identified a tridentate hexanuclear complex of arsenite on maghemite and magnetite nanoparticles by X-ray absorption spectroscopy (XAS). In addition, Farrell^36^ showed the thermodynamic stability of the tridentate arsenate complex using a density functional theory (DFT) calculation and revealed that the As-Fe distance was 2.41 Å in a tridentate complex with one hydroxide cluster.

Therefore, the objectives of this study were to evaluate the stability of goethite, examine the possibility of an arsenate tridentate complex and identify the effects of drying, pH and surface loading on the stability and structural configuration of arsenate on nano-goethite. To do so, we confirmed the structural transformation of nano-goethite with environmental changes, determined the structural configuration of arsenate on the nano-goethite surface including a tridentate complex, and simulated the effects of pH and surface loading on arsenate complexation using a macroscale batch experiment and nanoscale high-resolution transmission electron spectroscopy (HRTEM) and XAS measurements with DFT calculations.

## Results and Discussion

### Sorption isotherm and aqueous iron concentration

**Figure 1:**
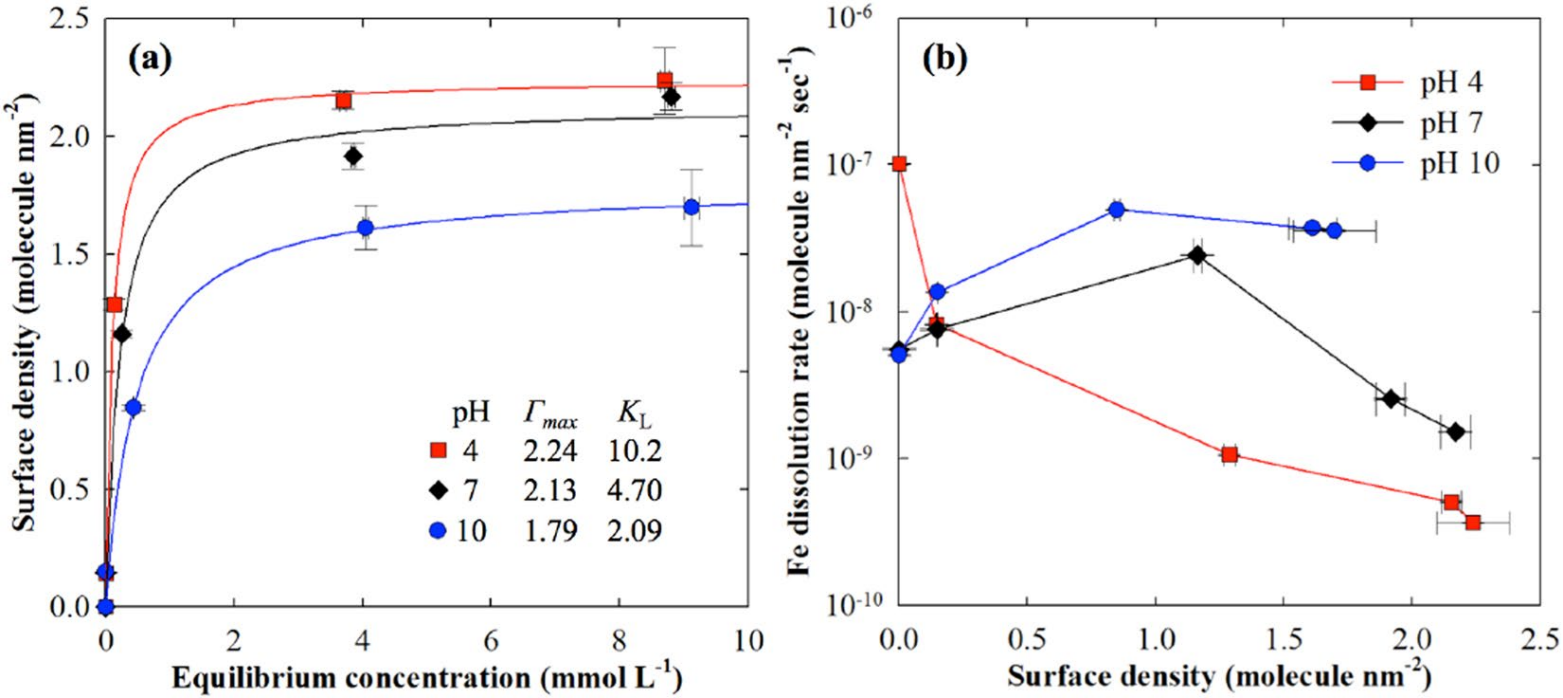
Langmuir sorption isotherms of arsenate (0, 0.1, 1, 5 and 10 mM initial concentrations) on nano-goethite plotted with surface density at pH values of 4 (red), 7 (black) and 10 (blue) (**a**). *Γ*_*max*_ and *K*_*L*_ are the maximum surface density and Langmuir coefficient, respectively. Scatterplot between surface density and Fe dissolution rate (molecule nm^−2^ sec^−1^) is used to explain the co-presence of arsenate and pH effect on Fe dissolution rate (**b**). Error bars indicate the standard deviations of three replicates.

The adsorption of arsenate on the goethite was measured using varying initial arsenate concentrations (0, 0.1, 1, 5 and 10 mM) and pH values (4, 7 and 10) for 48 h. We fitted the experimental data with Langmuir isotherms, and the result is illustrated in Fig. 1a. As a result, we found that the maximum adsorption capacities (*Γ*_*max*_) at pH values of 4, 7 and 10 were 2.24, 2.13 and 1.79 site nm^−2^, respectively, while the Langmuir constants (*K*_L_) were 10.2, 4.70 and 2.09 L site^−1^, respectively; the adjusted coefficients of determination were 0.994, 0.978 and 0.992 at pH values of 4, 7 and 10, respectively. The increase in pH resulted in the desorption of arsenate, and the difference in *Γ*_*max*_ dramatically increased with the change from pH 7 to 10 compared to that from pH 4 to 7. The values were 1.49, 2.31 and 1.43 site nm^−2^, respectively, which were consistent with those observed in previous studies. We also found that *K*_L_ dramatically decreased with increasing pH to values of 10.2, 4.70 and 20.9 L site^−1^ at pH values of 4, 7 and 10, respectively. The difference in *K*_L_ was higher with the change from pH 4 to 7 than from pH 7 to 10. Based on our results, we concluded that pH significantly decreased *Γ*_*max*_ and *K*_L_, which implied that fewer surfaces were available and less energy was favourable for arsenate sorption.

Assessing the dissolution rate of iron (DR_Fe_) with or without arsenate yielded interesting results (Fig. 1b). We observed a decrease in DR_Fe_ with increasing arsenate at a pH of 4, which was caused by inner-sphere (IS) complexation. Once the IS complex forms at the surface, the complex needs more energy to dissolve iron from goethite; therefore, DR_Fe_ decreased with increasing surface density in arsenate^15,37^. The result showed a decrease in DR_Fe_ with increasing pH without arsenate, which corresponded to that observed in previous studies^17,38^, but the DR_Fe_ pattern completely differed with arsenate at pH values of 7 and 10. There was a maximum DR_Fe_ at 1 mM arsenate; it gradually decreased at a pH of 7 with increasing arsenate, with no significant decrease observed at a pH of 10. We hypothesize that there are two possible mechanisms that may explain this change: the formation of an aqueous arsenate-ferric ion complex and the formation of a few nanosized iron precipitates with high surface charge. To test these hypotheses, we used a UV-Vis spectrophotometer to determine the arsenate-ferric ion complex based on a previous study^39^, and we confirmed the arsenate-ferric ion complex by measuring the absorbance peak at 280 nm at pH values of 4 and 7 (data not shown). However, we could not find the arsenate-ferric ion complex in the aqueous phase at a pH of 10, but we observed a background increase in the visible range, which indicated the formation of a crystal structure; thus, we utilized HRTEM to confirm the formation of nanosized iron precipitates.

### Transformation at pH 10 with arsenate

**Figure 2:**
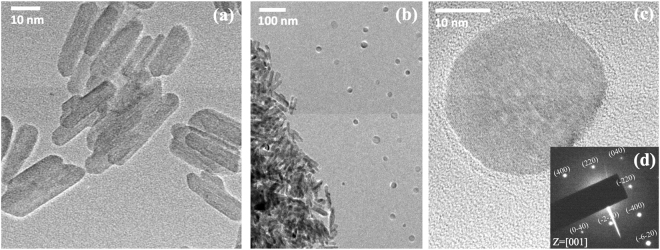
HRTEM image of rod-shaped goethite (**a**) and spherical bernalite by transformation from goethite (**c**), their distribution at a pH of 10 with 10 mM arsenate concentration (**b**), and selected area electron diffraction pattern of spherical bernalite at Z = [001] (**d**). We only found bernalite at a pH of 10 with arsenate concentration, and the abundance of bernalite increased with the arsenate concentration.

We measured HRTEM for all treatments containing arsenate (0–10 mM) and pH (4–10), and we observed three distinctive features in the treatments: square-shaped, rod-shaped and spherical particles. To identify each structure, we measured its selected area electron diffraction (SAED) pattern and element composition using HRTEM-EDS. As a result, we identified square-shaped particles as NaCl precipitates, as the EDS showed an approximately equal atomic ratio of Na and Cl with high abundances, and the sizes of the square-shaped particles varied from approximately 30–200 nm. We classified the rod-shaped particles as goethite (Fig. 2a) because the HRTEM image was identical to the specification sheet from the manufacturer, and the element composition of iron and oxygen was Fe_1_O_2.05_, which matched the composition of goethite. The size of goethite was 10.8 (±2.1) × 50.3 (±11.9) nm (n = 151), and we could not detect a significant decrease in size in all treatments; however, we observed an increase in DR_Fe_, which implied that the goethite had dissolved into ferric ions, but the level of DR_Fe_ did not lead to a significant difference in size because the dissolved ferric ions from the goethite ranged in size from to 0.38 × 10^−4^% to 1.1 × 10^−2^%. We also found that the degree of aggregation among the nanosized goethite increased with increasing pH and arsenate concentration, but we were not able to quantify the aggregation in this experiment.

We assumed that the 48-hr incubation time would not yield any precipitation or transformation under most conditions, but we observed spherical nanoparticles with diameters of 27.3 (±5.2) nm (n = 158) in the treatments with a pH of 10 and arsenate concentrations (1–10 mM). We found that the abundance of spherical nanoparticles increased with arsenate concentration, and we could not find any nanoparticles in treatments at a pH of 10 with low arsenate concentrations (0–0.1 mM). Based on these results, we presume that the spherical nanoparticles are dependent on arsenate and pH. Based on a review of the literature, we found several studies describing the transformation of goethite to other iron (hydr)oxide minerals in alkaline solution^16,40–42^. The inter-transformation among various iron (hydr)oxides is a well-known environmental phenomenon, and this transformation plays a key role in maintaining the ecosystem by retaining nutrients and pollutants and involving oxidation and reduction^15,16,43^. For that reason, we suspected that contamination occurred during the experiment or drying process. We purchased new reagents, repeated the experiment, and used a tightly sealed box for drying with purified ambient air; however, the spherical nanoparticle was still found at a pH of 10 with high arsenate concentrations. We measured the elemental composition using HRTEM-EDS, and the O/Fe ratios of the nano-goethite from the 10 mM arsenate treatment were 2.19 (±0.31), 2.15 (±0.15) and 2.10 (±0.42) at pH values of 4, 7 and 10, respectively, while that of the spherical nanoparticle was 3.20 (±0.27). Without arsenate, the O/Fe ratio was 2.05 (±0.09), and we concluded that presence of the arsenate complex on the surface was the reason why the oxygen concentration decreased on the goethite surface with increasing pH. However, a distinctive elemental composition was found in spherical nanoparticles at a pH of 10, which was approximately 1.5-fold higher than that of goethite. We also found trace levels (less than 1% atomic percent) of chromium, silica, and sulfur in all treatments, but these elements were also detected in nanoparticle-free spots with EDS; thus, we excluded their contributions to the spherical nanoparticles. In the HRTEM analysis, we observed large aggregates of rod-shaped goethite, but the spherical nanoparticle was only observed outside of the goethite aggregates, and the inter-particle distance was relatively constant without aggregation (Fig. 2b). This was hard to explain, but we assumed that its formation from aqueous ions occurred during the drying or inter-particle repulsion of previously formed nanoparticles due to the high surface charge. During the drying process, the water volume decreases, which increases the concentration of aqueous ions; thus, precipitation occurs, and numerous studies have reported a drying effect on precipitation^44,45^. However, in this case, because the goethite aggregate holds water longer due to its surface charge, the precipitated nanoparticle should form near the goethite aggregate. Ahn and Lee^46^ reported the formation of close-packed nanoparticles by partial drying, which can cause a constant distance without aggregation. Although both reactions could contribute to this phenomenon, it was impossible to identify the specific mechanism for the formation of spherical nanoparticles. To identify the crystal structure, we attempted to measure it using the fast Fourier transform function in the Digital Micrograph software (Gatan, USA). We obtained d-spacing values of 1.89, 2.69 and 2.71 Å from the spherical nanoparticle, but it was impossible to find the matched crystal structure from the reported transformation. We considered haematite the primary candidate because thermal dehydration easily transforms goethite to haematite *via* maghemite, and several studies have already discussed the formation of haematite^42,47^. Ralph *et al*.^16^ examined the effects of antimonate, arsenate and phosphate on the transformation of iron (hydr)oxides, and their results showed that arsenate and phosphate favoured the formation of haematite over goethite from ferrihydrite, but no transformation occurred at high concentrations (above 2.25 mM). In our laboratory, we also had 30-nm haematite purchased from US nano (US3160, USA), which had similar d-spacing values of 1.84, 2.51 and 2.70 Å, as determined by X-ray diffraction (XRD) analysis. However, the measured elemental composition and discordance in the [110] plane verified that the spherical nanoparticle was not haematite. By reviewing previous studies of minor abundant iron (hydr)oxides, we found that the elemental composition of bernalite (Fe(OH)_3_) was consistent with that of the nanoparticle^48^. To characterize its crystal structure, we employed SAED pattern analysis. Figure 2d shows the SAED pattern of spherical nanoparticles at the [001] axis. Based on the elemental composition and SAED pattern analysis, we confirmed that bernalite was the spherical nanoparticle. In addition, the EDS result showed that more arsenate was adsorbed onto the bernalite surface because the As/Fe ratios on goethite were 0.099, 0.093 and 0.083 for the treatments performed at pH values of 4, 7 and 10 in 10 mM arsenate, respectively, whereas the As/Fe ratio on bernalite was 0.181. This implied that bernalite had a higher sorption capacity per unit mass; however, a maximum value of only 0.0025% of the iron from the total iron weight (calculated from DR_Fe_) was assumed to be involved in the formation of bernalite nanoparticles. Thus, we ignored the effect of bernalite on the arsenate adsorption in further EXAFS measurements. However, the formation of bernalite in a real environment would be significant because of the colloidal transport in the presence of arsenate and high pH conditions.

### EXAFS and DFT results and linear combination fitting

**Figure 3:**
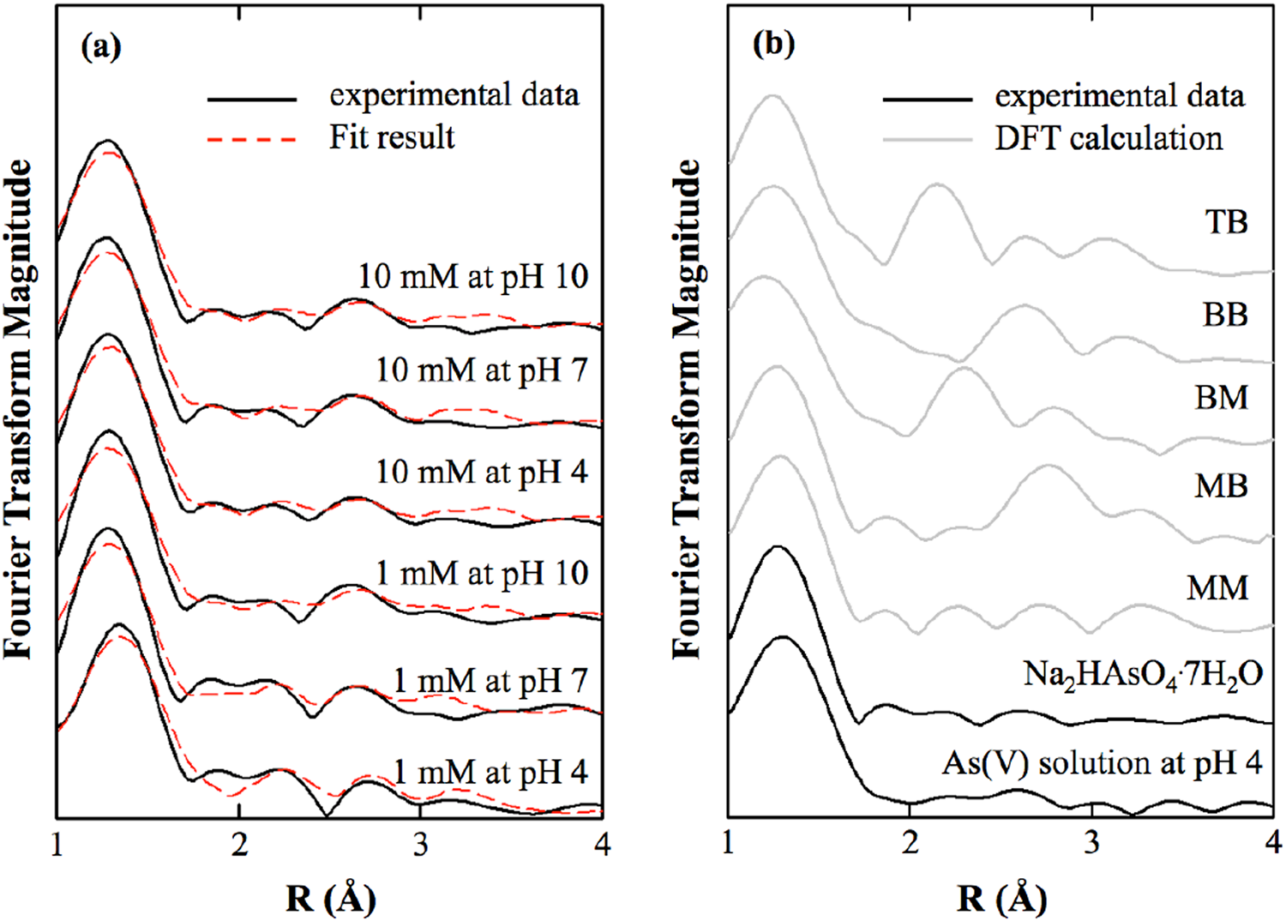
Fourier transform magnitude of As K-edge *k*^3^-weighted EXAFS spectra in sedimented samples at various pH values (4, 7, 10) and concentrations (1, 10 mM) (**a**), experimental spectra of arsenate solution and Na_2_HAsO_4_·7H_2_O powder as the references, and theoretical spectra of five configurations obtained by DFT calculations (**b**). The black line, red dotted line and grey line indicate the spectra of the experiment data, fit result and DFT calculation, respectively.

We attempted to measure the XAS spectrum for the 0.1, 1 and 10 mM arsenate concentrations at pH values of 4, 7 and 10 with dried and sedimented samples, but we could not measure the spectrum in the 0.1 mM treatment because we could not obtain a significant signal-to-noise ratio. We illustrate the experimental and theoretical As K-edge *k*^3^-weighted EXAFS spectra of sedimented and dried samples in Fig. 3 and Fig. S1, respectively. We also show the As K-edge *k*^3^-weighted spectra of the experimental and theoretical EXAFS in Fig. S2 and Fig. S3. We observed no difference in the X-ray absorption near edge structures (XANES) for all treatments (Fig. S4), which indicated that no change was observed in the oxidation/reduction status of arsenate. We also measured the spectra of the 10 mM arsenate solutions at pH values of 4 and 10 and the spectrum of the Na_2_HAsO_4_·7H_2_O reagent to identify the background of aqueous and precipitated arsenate. We used the aqueous and precipitated spectra as references for further linear combination fitting (LCF). We found slight differences in the aqueous arsenate spectra at pH values of 4 and 10, and there was a difference in the multiple scattering path changed by the symmetry of arsenate protonation (Fig. S2); thus, we merged the aqueous spectra at pH values of 4 and 10 to generate the aqueous spectrum at a pH of 7, where H_2_AsO_4_^−^ and HAsO_4_^2−^ existed at similar concentrations (pk_a2_ = 6.94). Based on the optimized geometry from the DFT, we calculated the theoretical spectrum of the BB, BM, MB, MM and TB complexes (Fig. S5). The M, B and T as the first letter indicate the dentation number, i.e., monodentate, bidentate and tridentate, respectively. The M and B in the second letter indicate the number of nuclei, i.e., mononuclear and binuclear, respectively. The first shell distance between arsenic and oxygen was 1.65–1.72 Å, which was consistent with the results of previous experiments and calculations. Based on these calculations, the atomic distances between the arsenic and two iron atoms were 3.59 and 6.00 Å for the MM complex, 3.33 and 3.35 Å for the MB complex, 2.72 and 4.70 Å for the BM complex, 3.21 and 3.34 Å for the BB complex, and 2.59 and 3.14 Å for the TB complex, respectively.

Our experimental results showed that more distinctive differences were found in the 1 mM treatment than in the 10 mM treatment, and we found significant shifts and transitions in the spectrum (Fig. S6); however, it was difficult to identify the specific sorption mechanism between the overlapping spectra. For that reason, we utilized the LCF with the theoretical spectra of five clusters and the experimental spectrum of aqueous and precipitated samples as the independent variables, and we set the spectrum of each treatment as the dependent variable. We also used the Fourier-transformed R space from 1 to 4 Å for the analysis because the EXAFS spectrum had slightly different features before the first shell due to the strong whiteline of arsenate. The Rbkg function of Athena software^49^ was not able to completely remove this effect. In addition, we only employed one arsenic atom and two iron atoms with several oxygen atoms for the DFT calculation; thus, this calculation did not have sufficient geometry for distances of more than 4 Å from the centre of the arsenic atom. The results of the LCF and analysis of variance (ANOVA) analyses are summarized in Table S2. The R-factor was 0.00007–0.00585 for sedimented samples and 0.00027–0.01725 for dried samples.

**Figure 4:**
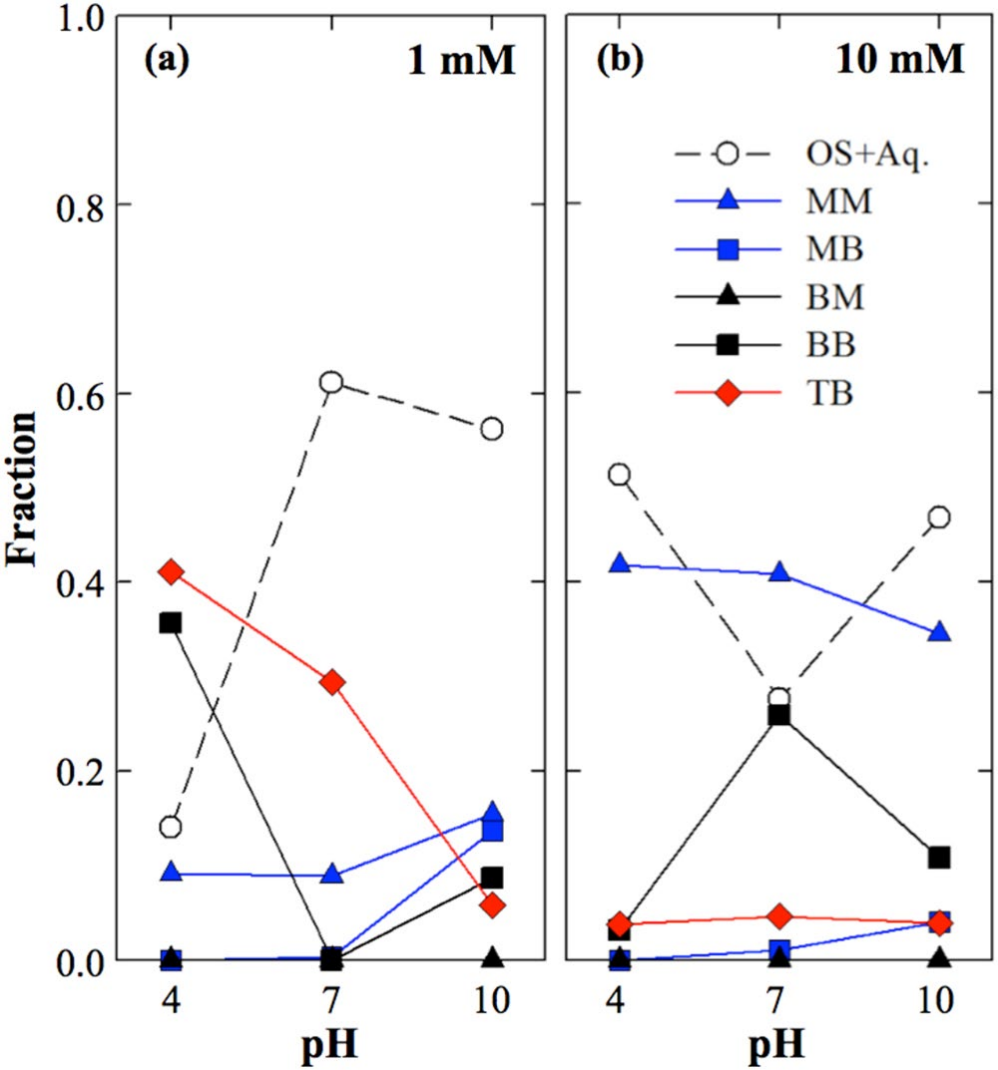
Distribution of five configurations of the complex by pH and arsenate concentration. The fraction results from the LCF and implies the relative abundance of each configuration, but not the mean concentration. White, blue, black and red indicate dentation status, and the circle, triangle, square and diamond represent the degree of nucleation.

The LCF results of the sedimented and dried samples are illustrated in Fig. 4 and Fig. S7 and listed in Table S2. These results show that the distribution of the complex changed with pH and arsenate concentration. The fraction indicates the relative abundance of each complex. However, the fraction did not indicate the concentration because we had already normalized the EXAFS spectrum and because we also used different sample thicknesses and densities for the measurement. Thus, the resulting fraction only implied the relative distribution of complexes. We used six independent variables: five from the DFT calculation and one from the arsenate solution experiment. We denoted the spectrum of arsenate solution as OS + Aq. because we could not separate the outer-sphere complex from the aqueous arsenate using EXAFS. In the dried sample, we used the precipitate instead of OS + Aq.

### pH and surface loading effects on structural configuration

First, we tried to determine the pH and surface loading effects. In the 1 mM treatment, we found significant increases in the OS + Aq., MM and MB complexes with increasing pH, but the TB and BB complexes decreased with increasing pH, while no BM complex was observed. In the 10 mM treatment, the TB and BM complexes showed no significant change with pH at low fractions, while the MM complex was dominant over all pH ranges, and the MM complex slightly decreased at a pH of 10. Interestingly, the OS + Aq. and BB complexes showed the opposite distribution with increasing pH, and the BB complex showed a maximum fraction at a pH of 7. We observed dramatic transitions in the structural configuration with both pH and arsenate surface loading.

Obviously, we identified, for the first time, the formation of the TB complex. As mentioned above, we observed the peak shifting at 2.5–2.7 Å, and we initially identified the peaks from the multiple scattering of As-O-O-As based on previous studies^18,50,51^. However, we looked up the normalized spectrum and found significant peak shifts in the 1 mM treatments, as well as less fluctuation in the 10 mM treatments (Fig. S4). We carefully reviewed numerous papers and we found that a few theoretical studies have reported the possibility of a tridentate complex between arsenate and iron (hydr)oxide. Farrell^36^ discussed the higher thermodynamic stability of a tridentate complex compared to that of a mono- or bidentate complex, and Waychunas^25,52^ suggested a face-sharing tridentate complex with a similar As-Fe distance as a bidentate complex. In addition, we also found several studies that identified a tridentate complex^34,35,53,54^ but not identify the arsenate. Thus, we included the tridentate complex for the DFT calculation and statistical analysis and found significant abundances of the TB complex in the treatment at pH values of 4 and 7 with 1 mM arsenate.

The fraction of OS + Aq. was relatively low at a pH of 4 and increased at a pH of 7 under the 1 mM treatment; then, it decreased at a pH of 10 (Table S2). The batch experiment revealed that only 57, 54 and 47% of surface sites were occupied (*Γ*/*Γ*_*max*_) and 13, 25 and 43% of arsenate remained in the solution phase at pH values of 4, 7 and 10, respectively. The difference between the fraction from the EXAFS study and the aqueous arsenate concentration from the batch experiment was mainly caused by the outer-sphere complex, which was not distinguishable by EXAFS, and numerous studies reported that the decrease in the OS complex with increasing pH was due to the change in the surface charge. Thus, the decrease in the IS complex led to a dramatic increase in OS + Aq. from a pH of 4 to 7, while a decrease in the OS complex caused a decrease in OS + Aq. from a pH of 7 to 10. Interestingly, we observed a sudden decrease of OS + Aq. with an increase in the BB complex at a pH of 7 under the 10 mM treatment. We assumed that both H_2_AsO_4_^−^ and HAsO_4_^2−^ are dominant at a pH of 7 and that the point of net zero charge (PNZC) of goethite was 5.66; thus, the combination of arsenate speciation and surface charge might lead to this result. In addition, we observed similar adsorption at pH values of 4 and 7 in the 10 mM arsenate treatment in the batch experiment, and the ferric arsenate complex or surface precipitation may have caused the formation of the BB complex.

The MM complex was observed at all treatments but was mainly found in the 10 mM arsenate treatments. The MM complex in the 1 mM treatment increased with increasing pH, but the effect of pH was the opposite in the 10 mM treatment. Recent studies have reported that surface loading determines the complex; Waychunas *et al*.^25^ initially reported the presence of an MM complex and a surface loading effect; Elzinga and Sparks^30^ also reported a surface loading effect using ATR-FTIR, and Abdala *et al*.^32^ reported that the MM complex of phosphate had high surface loading, while the BM and BB complexes were found to have low surface loading. For that reason, we explained that more competition at the limited sorption site led to the formation of the MM complex. A high concentration of arsenate caused more competition for the sorption site, and increasing pH changed the positively charged surface of goethite to a neutral or negatively charged surface, which could have limited the available sorption sites. As a result, both surface loading and pH caused the formation of the MM complex.

### Drying effect on structural configuration

Unlike in the sedimented samples, the LCF results of the dried samples showed relatively higher abundances of arsenate precipitate in all treatments. The precipitate fraction was calculated as 0.60–0.92, and the 1 mM treatment at a pH of 10 showed the minimum value of the precipitate fraction, while the 10 mM treatment at a pH of 10 showed the maximum value. The fractions of precipitate in the 1 mM treatment were 0.891, 0.866 and 0.600, while the fractions of precipitate in the 10 mM treatment were 0.899, 0.906 and 0.919 at pH values of 4, 7 and 10, respectively. We observed the opposite trend in the precipitate fraction, which we explained based on the batch experiment. In Table S2, the aqueous As contents in the 1 mM treatment were 0.661, 1.25 and 2.16 at pH values of 4, 7 and 10, respectively, while the adsorbed As contents were 44.8, 40.4 and 29.6 μmol, respectively. When we dried out the sample (0.25 g) with 5 mL of solution, the precipitate fractions at pH values of 4 and 7 were increased by the precipitation from aqueous arsenate and outer-sphere complex, but the outer-sphere complex of arsenate at a pH of 10 was dramatically decreased by the repulsion from the negatively charged surface, while a small amount of aqueous arsenate was precipitated; thus, the fraction of the precipitate was significantly decreased. As a consequence, the fraction of the inner-sphere complex was increased. In the 10 mM treatment, the aqueous As content was dramatically increased (21.1–65.8 times) from the 1 mM treatment, and the precipitate fraction was more than 0.899. We assumed that the amount of the desorbed outer-sphere complex from pH values of 4 to 10 was less than the amount of increased aqueous arsenate; thus, the fraction of the precipitate increased with increasing pH.

However, the fraction of the inner-sphere complex was too low to discuss the distribution of the structural configuration, and we also only employed Na_2_HAsO_4_·7H_2_O as the precipitate; thus, it was insufficient to explain the possible structural configuration and precipitation in the arsenate- and iron hydroxide-containing system. For example, we only employed di-sodium arsenate, but the precipitate can contain mono-sodium at low pH or tri-sodium at high pH. In addition, ferric arsenate could be precipitated in various atomic ratios and configurations, such as bernalite, which was confirmed by its formation at high pH with the presence of arsenate in this study. For that reason, our approach using Na_2_HAsO_4_·7H_2_O as the only precipitate may not be sufficient to explain the drying effect; however, employing as many reference precipitates as possible in the LCF would yield more explainable data in the future.

### Environmental implications

**Figure 5:**
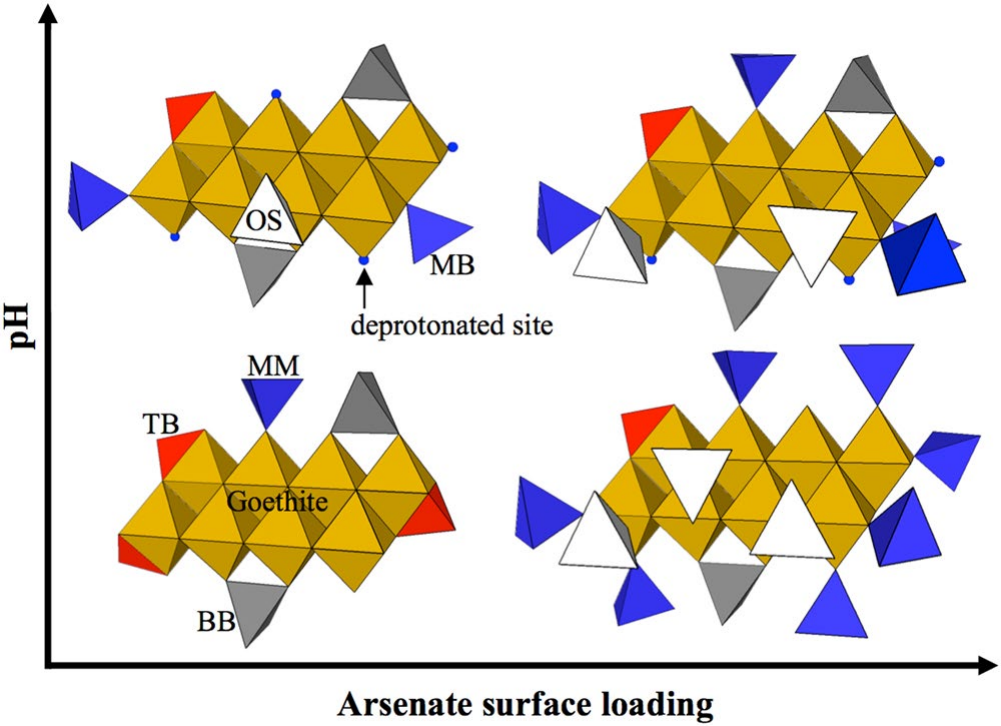
Schematic illustration of the transition of structural configuration with the change in pH and surface loading conditions. The white tetrahedral indicates the outer-sphere complex, while the blue, grey and red tetrahedra represent the monodentate, bidentate and tridentate complexes, respectively. The brown octahedral sheet represents the goethite.

In this study, we evaluated the structural configuration of arsenate with changes in concentration, drying and pH using a batch experiment and HRTEM and EXAFS measurements with the DFT calculation. We confirmed the formation of nanosized bernalite only in the presence of arsenate at a pH of 10 using HRTEM measurements, and the bernalite showed a 46% increase in its As/Fe ratio compared to the nano-goethite at a pH of 10. However, the formation of bernalite may enhance the colloidal transport of arsenate in wastewater treatment using iron (hydr)oxide or in a pH-increasing amendment-treated soil environment. In addition, we found that the TB and BB complexes were predominant at low pH and low surface loading conditions, while MM and MB were observed at high pH and high surface loading conditions (Fig. 5). The density of available sorption sites gradually decreases with increasing pH, and bidentate and tridentate complexes are predominant with less competition at low surface loading conditions because there are plenty of neighbouring sorption sites. However, the increase in pH or surface loading decreases the available sorption sites or the available sorption sites per arsenate; thus, the MM complex is dominant with the high competition at high surface loading conditions because there are not enough sorption sites to occupy.

These results may help us understand the effect of environmental conditions on the structural configuration of arsenate at the goethite-water interface. However, because this result only employed a single arsenate and two octahedral ferric hydroxides as the model sorbates for the DFT calculation, there is a significant discordance from reality. Employing a more detailed nano-goethite structure may lead to a more accurate interpretation. In addition, a denser experimental setup would yield a more persuasive result for this experiment.

## Materials and Methods

### Physiochemical characterization

A rod-shaped nano-goethite (US3162) crystal was purchased from US Research Nanomaterials (USA). To understand its crystal structure, we employed XRD using a D8 Advance (Bruker, Germany) with Cu Kα radiation from 5 to 90°. The XRD spectrum was compared to previous data from the American Mineralogist crystal structure database^55^. The (PNZC) was measured using the drift method^56^, and pH was potentiometrically measured using Orion 5 Star (Thermo, USA) in 1:200 (W/V). A Brunauer-Emmett-Teller (BET) isotherm with N_2_ gas was applied to measure the surface area using ASAP 2010 (Micromeritics, USA) at 77 K. All characteristics of nano-goethite are summarized in Table S1, and the XRD diffractogram is illustrated in Fig. S8.

### Batch experiment

To evaluate the adsorption capacity of arsenate on the goethite surface, we employed batch experiments at various pH and arsenate concentrations. Briefly, 50 mL of arsenate solution at different concentrations (0, 0.1, 1, 5 and 10 mM) were mixed with 0.25 g of dried goethite in a 50 mL C-tube. All solutions contained 0.1 M NaCl to maintain ionic strength. We employed the Langmuir isotherm to characterize the sorption behaviour of arsenate on the nano-goethite with varying pH values (4, 7 and 10), and we also conducted an iron dissolution experiment with or without arsenate concentrations. After 48 h of incubation using a vertical shaker (Daehan, Korea) at 200 rpm at room temperature, we transferred the 10 mL samples to the 15 mL C-tube for further XAS analysis. We centrifuged the samples at 4,200 RCF for 1 h, and the 35 mL of supernatant was filtered with a 0.2-μm PTFE syringe filter (Advantec, Japan). The supernatant readily acidified, and we used inductively coupled plasma optical emission spectroscopy (ICP-OES, Icap-7200, Thermo, USA) to measure the arsenic and iron concentrations in the solution. The centrifuged solid samples were oven-dried at 105 °C (24 h) before XAS measurements. The adsorbed arsenate was calculated by subtracting the initial amount from the aqueous amount. This procedure is schematically illustrated in Fig. S9. All reagents were purchased from Sigma-Aldrich (USA) with at least 98% purity.

### High-resolution transmission electron microscopy

The shape, size, crystal structure and elemental composition of the nano-goethite at each treatment were analysed by high-resolution transmission electron microscopy (HRTEM) using a JEM-3010 (Jeol, Japan), and its digital images and elemental compositions were obtained using a Gatan digital camera and energy dispersive spectroscopy (EDS), respectively. We placed one drop of sample from the XAS sample (without acidification) into a carbon film on a copper grid, and it dried overnight in a dust-free chamber. The sample grid was placed in a 60-mm Petri dish (SPL, Korea) and sealed with Parafilm for subsequent analysis. We employed Digital Micrograph software (Gatan, USA) to analyse the shape and size calculations, and we used CrysTBox software (Institute of Physics Academy of Science, Czech Republic) to interpret the SAED pattern^57^.

### X-ray absorption spectroscopy measurement

Synchrotron-based XANES and EXAFS measurements were performed on the 7D beamline of the Pohang Accelerator Laboratory (PLS-II, 3.0 GeV, 360 mA). The XAS spectra were collected in transmission and fluorescence modes for the dried samples but were only collected in fluorescence mode for the sedimented samples. We employed a Si(111) double crystal monochromator at room temperature with helium purging, and we measured the K-edge of As at 11.867 keV. We employed the dried and sedimented samples to evaluate the effect of drying on the structural configuration. The dried samples were ground for sample loading with Kapton tape, and we used a handmade holder for the sedimented samples, which has an inside volume of 5 (width) × 20 (height) × 10 (depth) mm; we injected aqueous samples. The surface was sealed with Kapton tape, and the samples were sedimented for 1 h before the XAS measurement. We used a sedimented goethite volume that was greater than the beam size, which was approximately 1 × 4 mm. We used NaH_2_AsO_4_·7H_2_O as the reference materials and measured 10 mM of aqueous arsenate at pH values of 4 and 10 for the background. We employed the Demeter software package (version, 0.9.25)^49^ for the normalization and background correction, and the spectra were converted to frequency (*k*) space weighted by *k*^3^. The *k*^3^-weighted spectra were Fourier-transformed to the R space using a Hanning window with *k* ranging from 3–11 Å^−1^. The structural configurations from the DFT calculations were employed for the FEFF calculations using FEFF8.5 lite^58^ to obtain the theoretical EXAFS spectra.

### Density functional theory calculation

All calculations were conducted using the DFT in Gaussian 09 software on a Tachyon 2 supercomputer at the supercomputing centre of the Korean Institute of Science and Technology Information (KISTI). The input file was generated with Avogadro software^59^. To calculate the geometry of arsenate on nano-goethite, we employed the density functional theory method B3LYP with the 6–311+G* basis set. The nano-goethite was simulated with a dual octahedral configuration of ferric hydroxide, which has been used in several DFT studies of arsenate binding for iron hydroxides^18,36,60^. We proposed five clusters for the calculation without considering the protonation of oxyanion and ferric hydroxide; thus, all clusters have a net charge of zero. We simulated the BB, BM, MB, MM and TB complexes with dual ferric hydroxides (Fig. S3).

### Statistical analysis

We used LCF instead of shell fitting; this reasons for this are: 1) We have no information about the scattering path of aqueous or precipitated arsenate, and it is difficult to predict the structure, but we have experimental EXAFS spectra of aqueous or precipitated arsenate; 2) There is a limitation on the variables to fit the spectra. We employed 5 DFT calculated structures with one aqueous or precipitated arsenate. We conducted shell fitting by assuming ΔE_0_ = 0 and σ^2^ = 0.0003 (to reduce the number of variables), which showed similar results as LCF, but the fitting result was not suitable because of the relatively high uncertainty with a high number of variables; 3) Working in R space allows us to selectively ignore higher coordination shells^61^. The measured spectrum from EXAFS and the calculated DFT spectrum were Fourier-transformed to R-space using the Athena software (Ravel and Newville, 2005)^49^, and the spectra were normalized and exported as CSV files within the R + ΔR range from 1 to 4 Å. We conducted LCF analyses to semi-quantify the distribution of the complex with changes in pH and concentration. The protocol for LCF was derived from the study of Paktunc (2004)^62^. We employed the Marquardt-Levenberg algorithm for the least-squares fit^63^. There are plenty of XAS data-analysis programs, such as SIXPACK^64^, Artemis^49^ and WinXAS^65^, which enable comprehensive data analysis, but these programs cannot address the complex mixed spectra in environmental and geological samples, which are complex mixtures in which it is essential to quantify the contribution of each component^62^. The fraction of the complex making up the experimental EXAFS spectrum is calculated by solving a mass balance equation^62^. We used Sigmaplot 10 (Systat, USA) to calculate the fraction in the LCF with the least-squares fit, and we constrained the fraction to >0 and employed 500 fits with 2,000 iterations.

## Electronic supplementary material

Supplementary Information
