# Supplementary material for: Identification of Bernalite Transformation and Tridentate Arsenate Complex at Nano-goethite under Effects of Drying, pH and Surface Loading
Source: Sci Rep. 2018 May 30;8:8369. doi: 10.1038/s41598-018-26808-4 (PMC5976659; doi:10.1038/s41598-018-26808-4)
Supplement: Supplementary file 1 — Supplementary Information [file 41598_2018_26808_MOESM1_ESM.docx]

Supporting Information

Identification of Bernalite Transformation and Tridentate Arsenate Complex at

Nano-goethite under Effects of Drying, pH and Surface Loading

Junho Han^1^, Hee-Myong Ro^1*^

^1^Department of Agricultural Biotechnology and Research Institute of Agriculture and Life Sciences, Seoul National University, Seoul, 08826 Republic of Korea

*To whom correspondence should be addressed

Phone: 82-2-880-4645

Fax: 82-2-873-3112

Email: hmro@snu.ac.kr

Number of pages: 12

Number of tables: 2

Number of figures: 9

**Table S1** Physiochemical characteristics of nano-goethite.

| Characteristics | Value |
| --- | --- |
| pH | 6.13 (0.17) |
| EC (uS cm^-1^) | 0.137 (0.01) |
| PNZC | 5.66 |
| SA (m^2^ g^-1^) | 83.9 |
| Shape | rod |
| Size (nm) | 50.3 x 10.8 |

EC electrical conductivity (μS cm^-1^); PNZC point of net zero charge; SA surface area (m_2_ g^-1^)

pH and EC were measured at 1:200 (g:mL).

PZC and SA were measured by the drift method and N_2_-BET method at 77 K, respectively.

The values in parentheses indicate the standard deviation of triplicates

**Table S2** Results from batch experiments and linear combination fitting of EXAFS spectra acquired in sedimented and dried samples.

| **Experimental condition** | | |  | **Batch experiment** | | | |  | **Linear combination fitting of EXAFS spectra** | | | | | | | |
| --- | --- | --- | --- | --- | --- | --- | --- | --- | --- | --- | --- | --- | --- | --- | --- | --- |
| Phase | Conc. (mM) | pH |  | Aqueous  As (μmol) | adsorbed  As (μmol) | *Γ* (N nm^-2^) | *Γ/ Γ_max_*x100 |  | Fraction | | | | | | | R-factor |
|  |  |  |  |  |  |  |  |  | MM | MB | BB | BM | TB | Solution | Powder |  |
| Sedimented | 1 | 4 |  | 6.61  (0.098) | 44.8  (0.786) | 1.29  (0.022) | 57.4% |  | 0.091  (0.052) | 0.000  (0.029) | 0.357  0.054) | 0.000  (0.035) | 0.411  (0.027) | 0.141  (0.043) | - | 0.00585 |
| Sedimented | 1 | 7 |  | 12.5  (0.142) | 40.4  (0.549) | 1.16  (0.016) | 54.5% |  | 0.090  (0.072) | 0.004  (0.042) | 0.000  (0.077) | 0.000  (0.049) | 0.295  (0.037) | 0.612  (0.060) | - | 0.00041 |
| Sedimented | 1 | 10 |  | 21.6  (0.179) | 29.6  (0.429) | 0.849  (0.012) | 47.4% |  | 0.156  (0.056) | 0.137  (0.033) | 0.087  (0.062) | 0.000  (0.039) | 0.058  (0.029) | 0.562  (0.047) | - | 0.00402 |
| Sedimented | 10 | 4 |  | 435  (3.75) | 77.9  (4.92) | 2.24  (0.141) | 99.9% |  | 0.418  (0.045) | 0.000  (0.026) | 0.000  (0.049) | 0.032  (0.032) | 0.038  (0.024) | 0.513  (0.038) | - | 0.00028 |
| Sedimented | 10 | 7 |  | 440  (3.28) | 75.6  (2.01) | 2.17  (0.058) | 102% |  | 0.408  (0.055) | 0.010  (0.032) | 0.000  (0.060) | 0.259  (0.038) | 0.046  (0.028) | 0.276  (0.047) | - | 0.00014 |
| Sedimented | 10 | 10 |  | 456  (5.57) | 59.1  (5.63) | 1.70  (0.162) | 94.8% |  | 0.345  (0.048) | 0.040  (0.028) | 0.000  (0.053) | 0.108  (0.035) | 0.040  (0.025) | 0.468  (0.040) | - | 0.00007 |
| Dried | 1 | 4 |  | 0.661  (0.010) | 44.8  (0.786) | 1.29  (0.022) | 57.4% |  | 0.000  (0.031) | 0.005  (0.016) | 0.070  (0.028) | 0.014  (0.022) | 0.019  (0.015) | - | 0.891  (0.053) | 0.01725 |
| Dried | 1 | 7 |  | 1.25  (0.014) | 40.4  (0.549) | 1.16  (0.016) | 54.5% |  | 0.003  (0.018) | 0.005  (0.009) | 0.069  (0.016) | 0.000  (0.012) | 0.057  (0.009) | - | 0.866  (0.031) | 0.00535 |
| Dried | 1 | 10 |  | 2.16  (0.018) | 29.6  (0.429) | 0.849  (0.012) | 47.4% |  | 0.174  (0.096) | 0.000  (0.048) | 0.098  (0.084) | 0.020  (0.066) | 0.109  (0.046) | - | 0.600  (0.162) | 0.00181 |
| Dried | 10 | 4 |  | 43.5  (0.375) | 77.9  (4.92) | 2.24  (0.141) | 99.9% |  | 0.000  (0.030) | 0.075  (0.017) | 0.027  (0.030) | 0.000  (0.022) | 0.000  (0.015) | - | 0.899  (0.049) | 0.00062 |
| Dried | 10 | 7 |  | 44.0  (0.328) | 75.6  (2.01) | 2.17  (0.058) | 102% |  | 0.000  (0.029) | 0.000  (0.015) | 0.060  (0.027) | 0.034  (0.022) | 0.000  (0.015) | - | 0.906  (0.048) | 0.00053 |
| Dried | 10 | 10 |  | 45.6  (0.557) | 59.1  (5.63) | 1.70  (0.162) | 94.8% |  | 0.000  (0.027) | 0.000  (0.013) | 0.037  (0.024) | 0.000  (0.018) | 0.044  (0.013) | - | 0.919  (0.046) | 0.00027 |

*Aqueous As* arsenate mol in aqueous phase after batch experiment measured by ICP-OES, where the difference in sedimented (50 mL solution) and dried (5 mL supernatant) samples is caused by the volume difference of the samples; *Adsorbed As* adsorbed arsenate on the goethite determined by subtracting the aqueous concentration from the total concentration; *Γ* (N nm^-2^) surface density; *Γ/ Γ_max_*x100 surface coverage percent calculated with Langmuir isotherm parameter; *MM* monodentate mononuclear complex; *MB* monodentate binuclear complex; *BB* bidentate binuclear complex; *BM* bidentate mononuclear complex; *TB* tridentate binuclear complex; The values in parentheses indicate the estimated standard error; *R-factor* = Σ(data-fit)^2^/Σ(data)^2^.

**
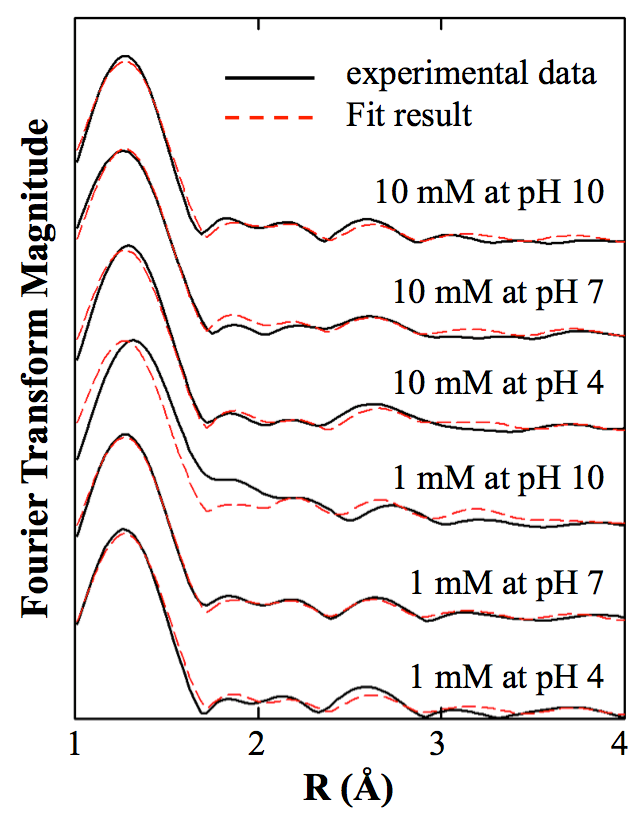
**

**Fig. S1** Fourier transform magnitude from As K-edge *k^3^*-weighted EXAFS spectra of dried samples under various pH values (4, 7 and 10) and surface loading (1 and 10 mM) conditions. The black solid lines and red dashed lines indicate the spectra of experimental data and fit result from linear combination fitting analysis, respectively.


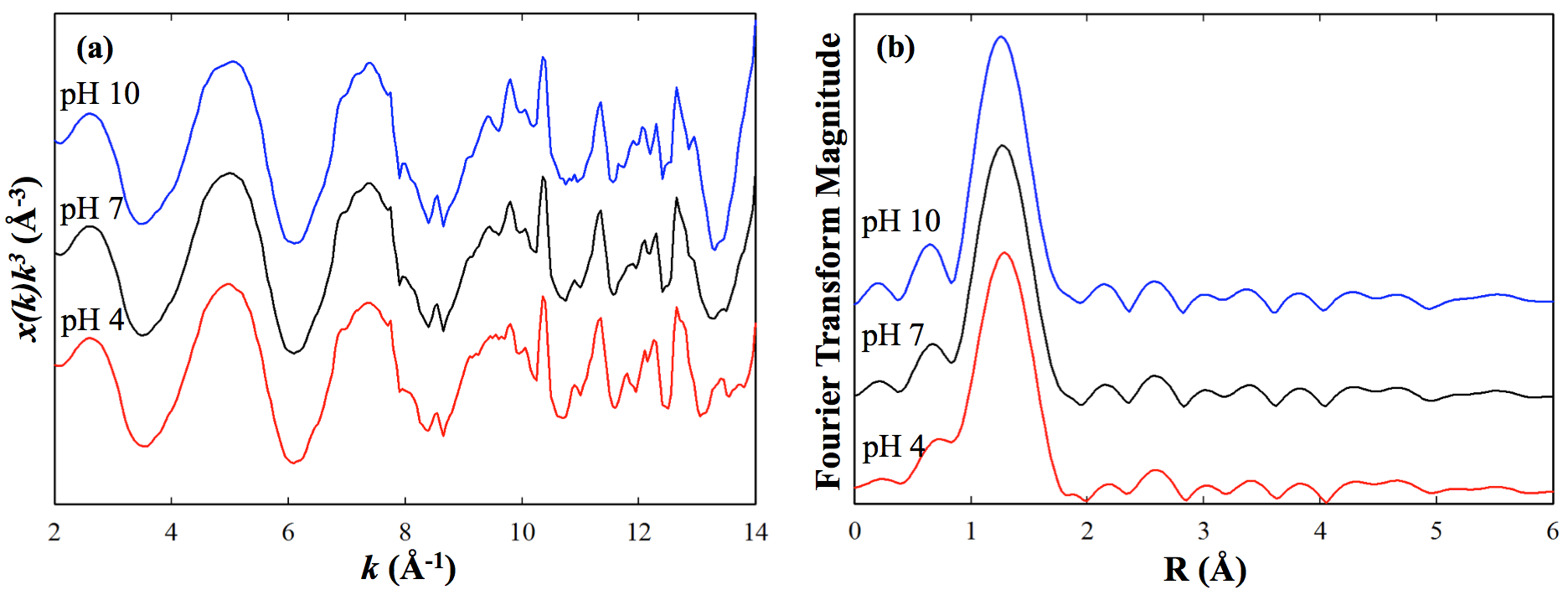


**Fig. S2** As K-edge k^3^-weighted EXAFS spectra (a) and the corresponding Fourier transform magnitude (b) of 10 mM aqueous arsenate at pH values of 4 (red), 7 (black) and 10 (blue). The 10 mM aqueous arsenate at pH values of 4 and 10 were measured by EXAFS, and the 10 mM aqueous arsenate at a pH value of 7 represents the merged experimental spectra of pH 4 and 10.

**
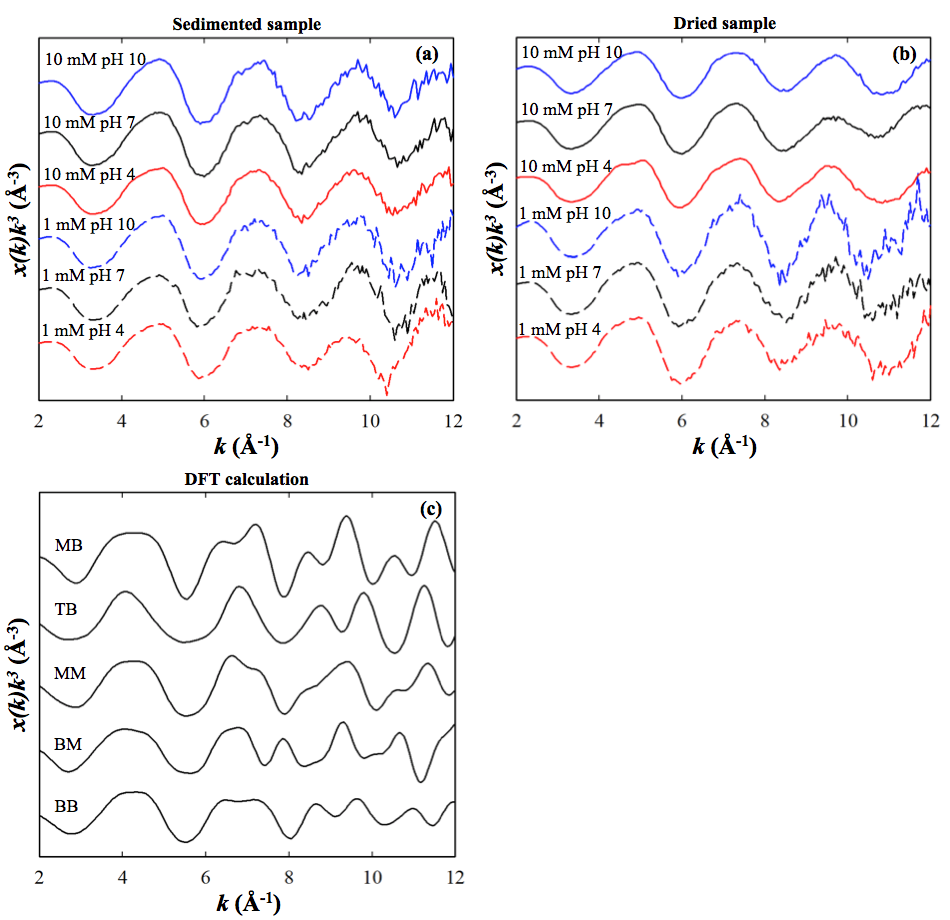

Fig. S3** Experimental As K-edge k^3^-weighted EXAFS spectra of 1 mM (dotted line) and 10 mM arsenate (solid line) on the goethite at pH values of 4 (red), 7 (black) and 10 (blue) in different sample phases (sedimented (top) and dried (middle)), and theoretical As K-edge k^3^-weighted EXAFS spectra of five clusters of the complex from the DFT calculation (bottom).


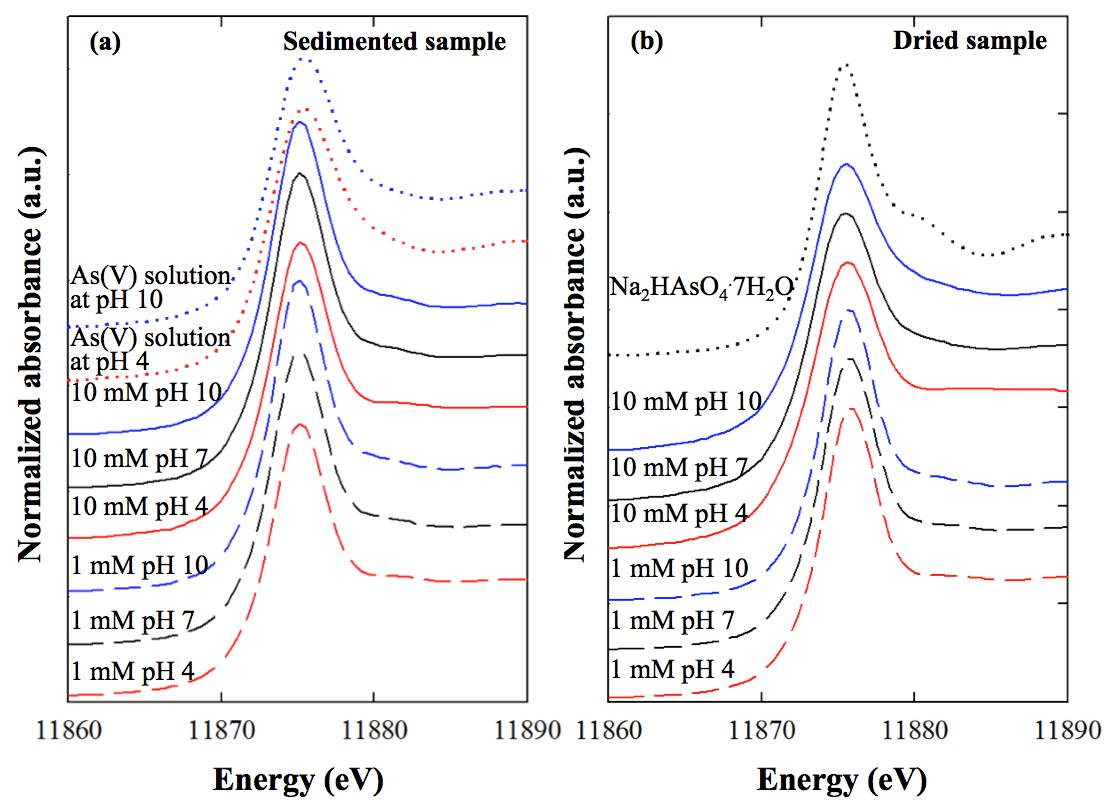


**Fig. S4** As K-edge XANES spectra of 1 mM (dashed line) and 10 mM arsenate (solid line) on the goethite at pH values of 4 (red), 7 (black) and 10 (blue) in different sample phases (sedimented (a) and dried (b)), and the aqueous arsenate (dotted line) and precipitate (dotted line).


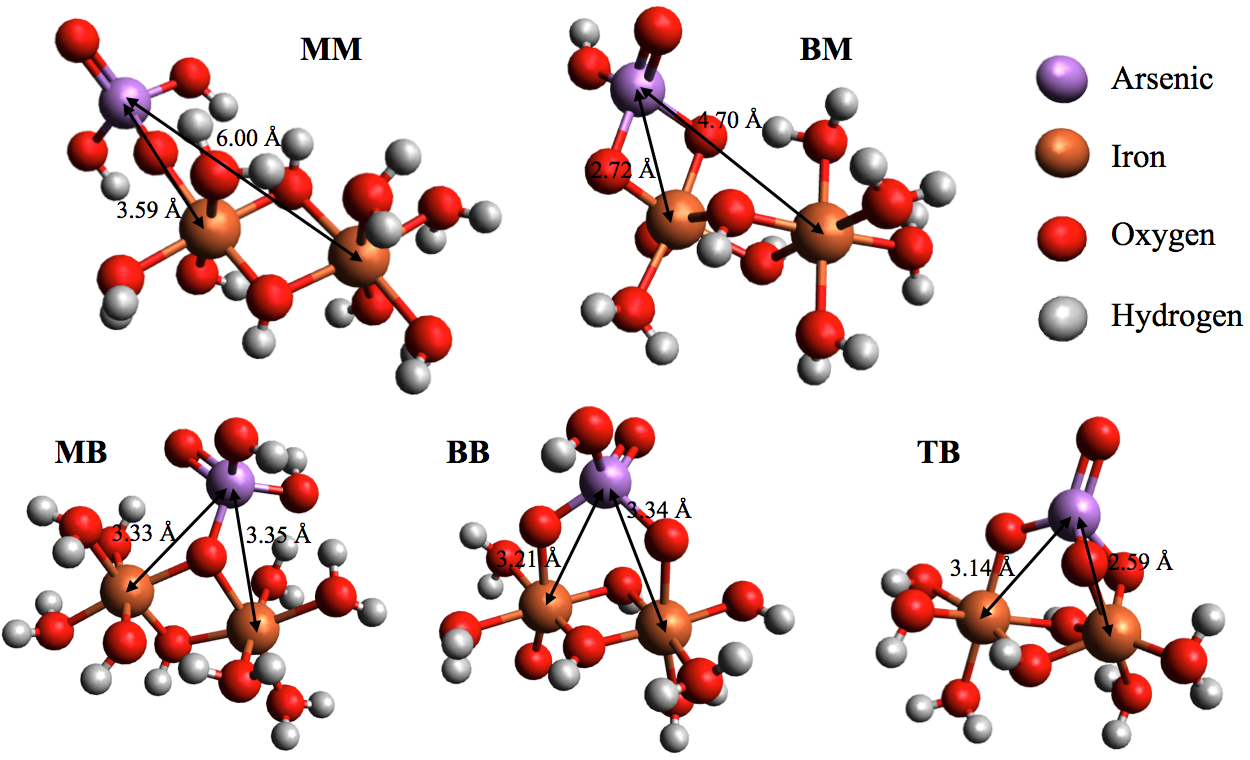


**Fig. S5** Optimized geometry by DFT calculations of B3LYP with the 6-311+G* basis set. The first letter of M, B and T indicates the dentation number, i.e., monodentate, bidentate and tridentate, respectively, while the second letter indicates the number of nuclei, i.e., mononuclear (M) or binuclear (B). The number indicates the interatomic distance between As and Fe.


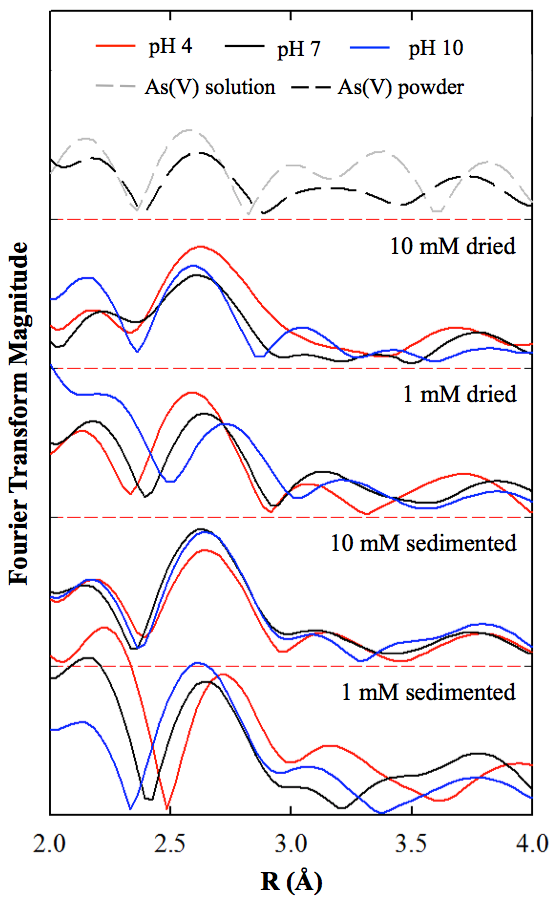


**Fig. S6** Fourier transform magnitude from As K-edge *k^3^*-weighted EXAFS spectra of sedimented and dried samples at pH values of 4 (red), 7 (black) and 10 (blue) at 1 and 10 mM arsenate surface loading. The dashed grey and dashed black lines are the spectra of the 10 mM arsenate solution at a pH of 4 and the Na_2_HAsO_4_^.^7H_2_O powder, respectively.


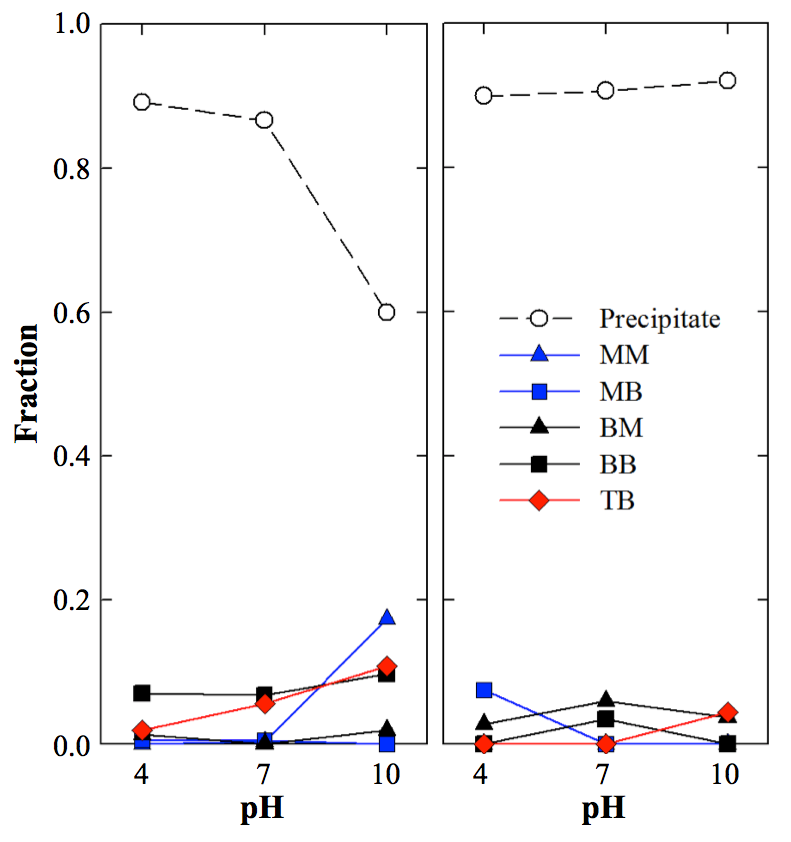


**Fig. S7** Distribution of five configurations of the complex and precipitated arsenate (using NaH_2_AsO_4_^.^7H_2_O) at pH values of 4, 7 and 10 and arsenate concentrations (1 and 10 mM) after drying. The fraction was calculated by linear combination fitting; it represents the relative abundance of each configuration, but not the mean concentration. White, blue, black and red reflect the number of dentation, and the circle, triangle, square and diamond represent the number of nucleations.


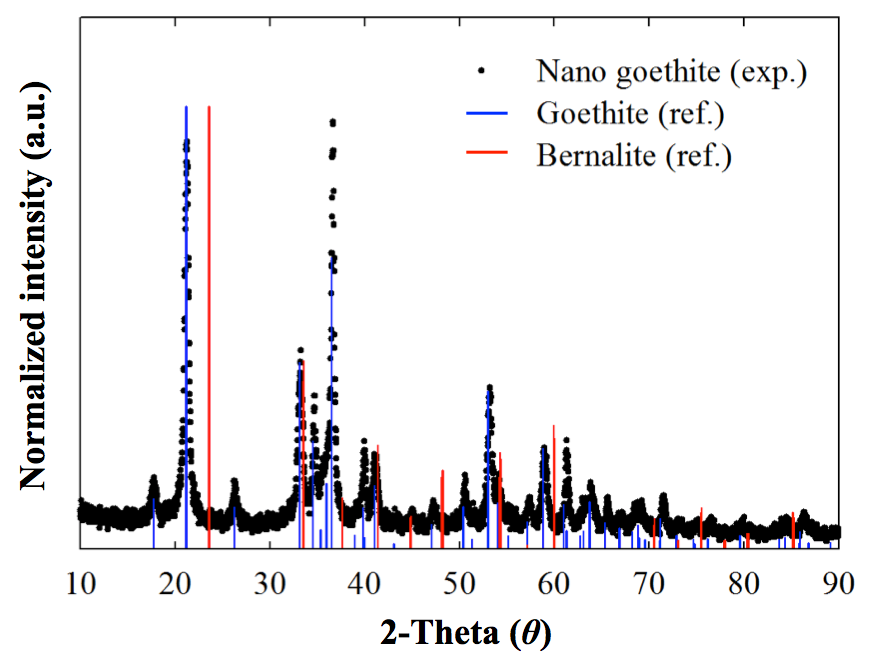


**Fig. S8** X-ray diffraction spectrum of the goethite sample (black dot) and reference goethite (blue bar, AMCSD-0003165) and bernalite (red bar, AMCSD-0001607) from the American Mineralogist crystal structure database.


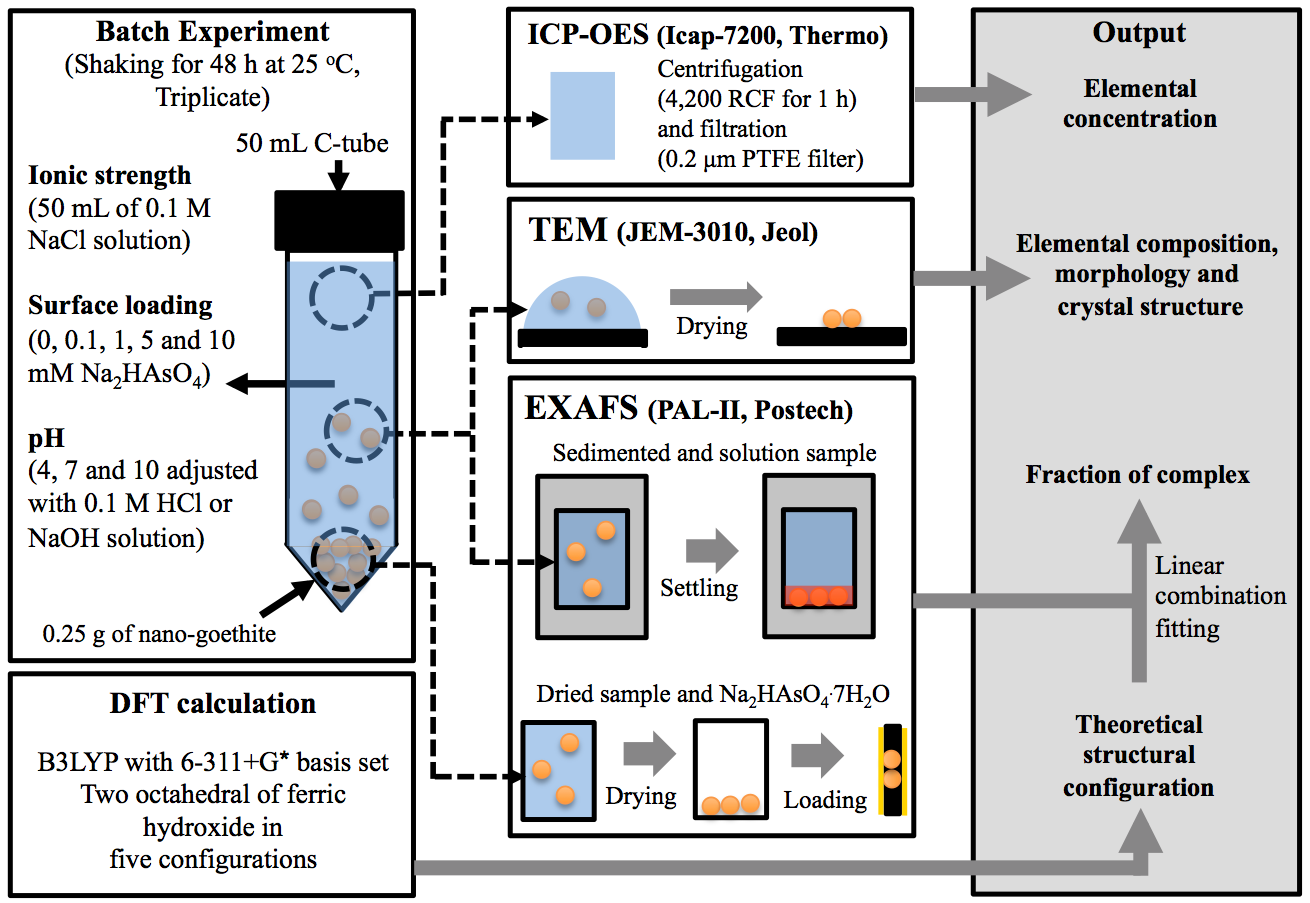


**Fig. S9** Schematic illustration of the batch experiment, DFT calculation, ICP-OES, TEM and EXAFS measurements and the interpretation of the output.
